# Integrated Metabolome and Transcriptome Analyses Reveal Dissimilarities in the Anthocyanin Synthesis Pathway Between Different Developmental Leaf Color Transitions in *Hopea hainanensis* (Dipterocarpaceae)

**DOI:** 10.3389/fpls.2022.830413

**Published:** 2022-03-03

**Authors:** Guihua Huang, Xuezhu Liao, Qiang Han, Zaizhi Zhou, Kunnan Liang, Guangyou Li, Guang Yang, Luke R. Tembrock, Xianbang Wang, Zhiqiang Wu

**Affiliations:** ^1^State Key Laboratory of Tree Genetics and Breeding, Research Institute of Tropical Forestry, Chinese Academy of Forestry, Guangzhou, China; ^2^Shenzhen Branch, Guangdong Laboratory for Lingnan Modern Agriculture, Genome Analysis Laboratory of the Ministry of Agriculture and Rural Affairs, Agricultural Genomics Institute at Shenzhen, Chinese Academy of Agricultural Sciences, Shenzhen, China; ^3^Guangdong Eco-Engineering Polytechnic, Guangzhou, China; ^4^Department of Agricultural Biology, Colorado State University, Fort Collins, CO, United States

**Keywords:** plant pigments, leaf maturation, trees, anthocyanins, transcription factors

## Abstract

Changes in plant leaf color during development are directly related to the accumulation or degradation of certain phytochemicals such as anthocyanins. Since some anthocyanins can be beneficial to human health and provide insights into the biology of leaves, the underlying processes and timing by which plants produce these molecules has been the focus of numerous studies. The tree species *Hopea hainanensis* generally produces green leaves at all growth stages; however, a few explored individuals have been identified possessing red leaves on the top of the seedlings at a young stage. While the phenomenon of leaf color varying with age has been studied in several species, the underlying mechanisms are largely unknown in *H. hainanensis*. Using a metabolomics approach, the young red leaves in *H. hainanensis* were found to contain higher levels of anthocyanins and flavonoids than the young green-leaved individuals. Among anthocyanins, pelargonidin and cyanidin were the most likely candidates contributing to the red color of the young leaves. Transcriptome results indicated the genes related to the production of these anthocyanins were significantly upregulated, leading to greater accumulation of red pigments. Specifically, the expression of several *MYB* and *bHLH* genes in young red leaf lines was significantly higher than that in the young green leaf lines, especially *HhMYB66*, *HhMYB91*, *HhMYB6*, and *HhbHLH70*. As such these four transcription factors are probably the main regulatory genes resulting in young red leaves in *H. hainanensis*. From these results, comparative analyses with other species can be made to better understand the evolution of pigment biosynthesis and how anthocyanins function in plant metabolism and evolution/adaptation.

## Introduction

Differences in leaf color have been observed by humans for millennia, especially in temperate climates where many trees reveal colorful pigments in autumn as chlorophyll is degraded and secondary pigments are made evident. Beyond the aesthetics of leaf coloration, pigment molecules play an essential role in leaf physiology and metabolism through harvesting light and providing protection from the damaging effects of UV radiation (Demmig-Adams and Iii, 1996; Chen, 2014; Rahnasto-Rilla et al., 2018). The protective effects of some plant pigment molecules, such as anthocyanins, are known to provide similar functions in human biochemistry, and thus a great deal of work has been conducted to better understand these molecules and how they can be used medicinally (Santos-Buelga et al., 2014; Demmig-Adams et al., 2020; Jideani et al., 2021). Therefore, improving our understanding of plant pigment biosynthetic pathways could have profound influences on both plant and human health. For example, the development of cultivars with specific leaf or fruit pigmentation could be improved by molecular breeding and/or genetic engineering of specific steps in pigment biosynthesis gene networks only after the pathways are properly mapped.

*Hopea hainanensis* Merr. et Chun (坡垒 po lei) is a tropical evergreen tree species in the Dipterocarpaceae family. The current range of this species is restricted to scattered occurrences in dense tropical forests on the island of Hainan and a small number of climactically similar locations in Northern Vietnam. This species is well-known due to the fine-grained durable hardwood it possesses, which has historically been used for a wide variety of applications such as in the production of railroad sleepers, mechanical appliances, fishing vessels, docks, bridges, and in constructing buildings. Since this species is now uncommon in the wild due to intensive logging and habitat loss (Ly et al., 2018), the uses of the wood are now mainly restricted to making decorative furniture. In addition to timber production, white aromatic resin from the trunk of *H*. *hainanensis* has been used in making medicine, perfume, and paint. The characterization of *H. hainanensis* for medicinal compounds has resulted in the discovery of several novel and bioactive compounds, including the acetylcholinesterase inhibitor hopeahainol (Ge et al., 2008, 2009). Moreover, *H*. *hainanensis* is a deep-rooted tree with tolerance to rocky and shallow soils making it highly valuable in ecosystem services related to soil and water preservation and as such it has been often used in landscaping and ecological conservation projects.

Most recent research on *H. hainanensis* has focused on conservation biology and ecology, seed germination, breeding, reintroduction, and silvicultural attributes (Xiaoying et al., 2017; Lu et al., 2020). Although some chemistry work has been conducted regarding the medical chemistry of *H. hainanensis* (Ge et al., 2008, 2009), relatively little work has been completed regarding the developmental biology of leaf pigment phytochemistry in this species.

In the course of previous studies on *H. hainanensis*, we found that some trees possessed green leaves through the developmental process, whereas others produced leaves that were initially red when first developing and eventually became green as the leaves matured. Breeding experiments between the two leaf forms indicated that the trait was heritable and as such likely not the results of a plastic gene by environment response. Given the desirable nature of this trait in the development of ornamental cultivars and the potential underlying adaptive characteristics associated with pigmentation (such as adaption to different light levels), it was determined that further work should be conducted to map the genetic pathways of these two color forms. Young red leaf forms have been studied in several plant species (Han et al., 2019; Waterland et al., 2019; El-Nakhel et al., 2020); however, it has not been characterized in *H. hainanensis*. Given this gap in knowledge, we carried out metabolome and transcriptome analyses of different colored leaves at different stages of development for *H*. *hainanensis* to answer the following questions regarding red leaf pigmentation: (1) what phytochemicals are responsible for the red coloration in young red leaves, (2) which genes are upregulated in the biosynthesis of red pigments, and (3) how do these attributes differ between the two young leaf forms and to other known pigment pathways? The results from these investigations will elucidate the mechanisms responsible for young red leaves in *H. hainanensis* and provide a thoroughly described trait for testing hypotheses regarding the ecology and evolution of the two leaf forms and improve our understanding of how increased red pigmentation in young leaves could influence other biosynthetic pathways especially as pertains to medicinal compounds, environmental adaptation, and photosynthetic efficiency. Young red leaf lines are considered desirable for horticultural uses, and thus knowledge about the formation of this trait can improve cultivar selection and could lead to the development of new more desirable and resilient cultivars for use in landscaping and ecological restoration. Except for cultivar characterization, the inclusion of different phytochemical variants into the germplasm repositories (especially among rare trees like *H. hainanensis*) is essential to preserve the diversity of traits for breeding and introduction.

## Results

### Metabolites in *Hopea hainanensis*

**FIGURE 1:**
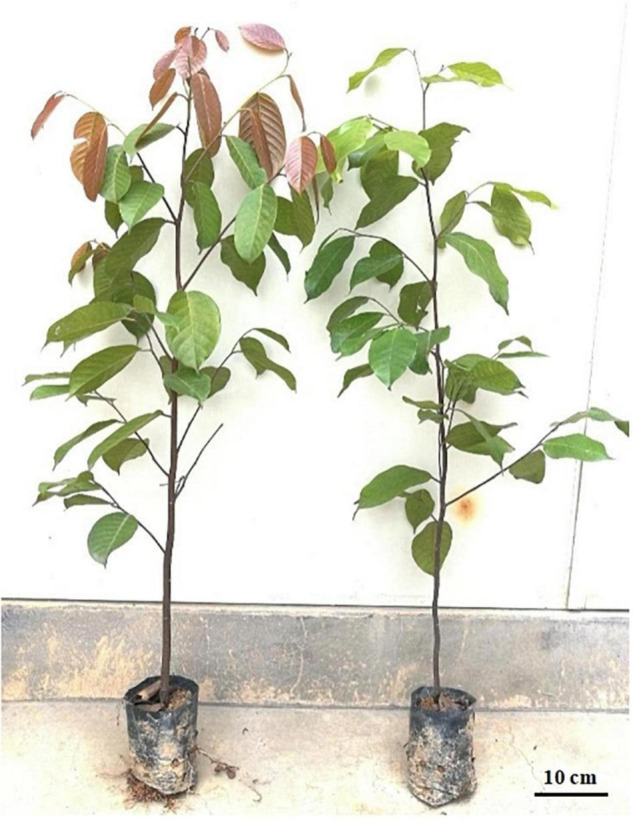
Different leaf color phenotypes in *Hopea hainanensis*. Young red-leaved example on the left and standard green-leaved example on the right.

*Hopea hainanensis* is an economically and ecologically important species in Dipterocarpaceae, which was found to possess young red-leaved individuals in about 10% of the individuals sampled (Figure 1).

**FIGURE 2:**
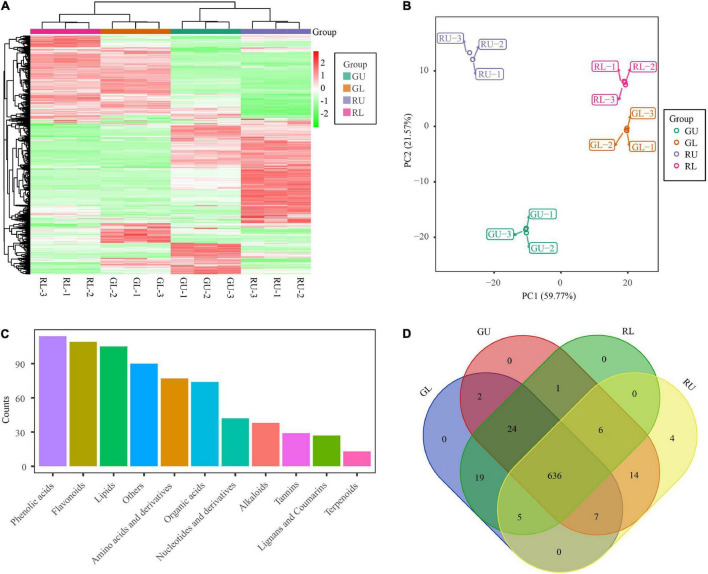
Metabolites of *H. hainanensis*. **(A)** Heatmap of metabolites distributed in all samples and replicates. **(B)** PCA analysis of metabolites. **(C)** Abundance of different metabolite types from all samples. **(D)** Venn diagram of metabolites from all samples.

To investigate what compounds contribute to the red-colored young leaves in *H. hainanensis*, the total metabolites of RU (red young leaves of red lines), RL (green mature leaves of red lines), GU (green young leaves green lines), and GL (green mature leaves of green lines) were detected by UPLC-MS/MS followed by hierarchical cluster analysis (HCA) to analyze the differences in accumulated metabolites of the different samples. The metabolites possessing similar accumulation patterns were clustered together and indicated that there are more differences in young leaves than mature leaves between the comparison of RU vs. GU and RL vs. GL. However, even in the mature leaves of the two lines, several metabolites were differentially accumulated. The HCA results also showed that some of the metabolites of RU were significantly different from those of the other three sample types. Nearly half of all detected metabolites were significantly accumulated in RU, some of which are likely contribute to red young leaves (Figure 2A).

In addition, the results of principal component analysis (PCA) with all samples showed that principal component 1 (PC1) and principal component 2 (PC2) accounted for 59.77 and 21.57% of the total variation, respectively. In the PCA, the biological replicates were tightly clustered while the four sample types were broadly separated on the graph. The RU samples were clearly differentiated from the other three samples, yet the mature leaves from the different lines were also plotted separately (and non-overlapping) in the PCA plot. These results not only indicate the repeatability and reliability of the data, but also indicate metabolic differences by stage and type across all samples (Figure 2B). To resolve the key metabolites responsible for the young red leaves in *H. hainanensis*, 719 metabolites were detected when all RU, RL, GU, and GL were considered. The most abundant compounds were phenolic acids (114), flavonoids (109), lipids (105), amino acids and derivatives (77), and organic acids (74), among which the flavonoids are considered the most important in this study because of their crucial role in plant pigmentation (Figure 2C). Of the 719 metabolites, 636 metabolites were shared across all four sample types, with only four metabolites (including flavonoids related to red color) unique to RU (Figure 2D), and no specific metabolites were uniquely accumulated in the other three samples. Given that flavonoids as well as several unique flavonoids are found in higher abundance in RU, it is reasonable to suggest that compounds of this type are involved with red coloration among these samples (Figures 2A,C,D).

### Differential Accumulation of Flavonoid Metabolites Between RU and GU

**FIGURE 3:**
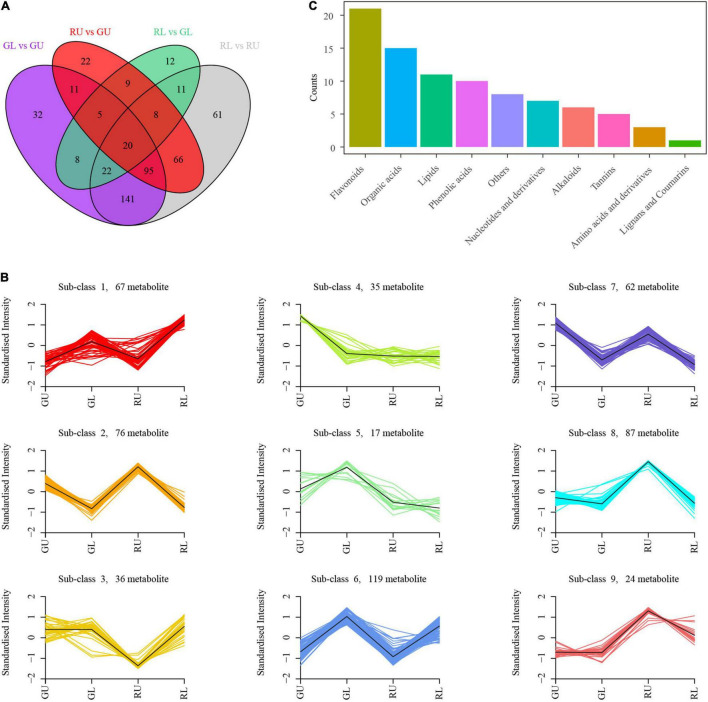
DAMs in *H. hainanensis*. **(A)** Venn diagram of DAMs from all comparisons. **(B)** k-means clustering analysis of total differential metabolites. **(C)** DAMs abundance by type in subclass 8.

To further confirm that flavonoid metabolites were responsible for the formation of red young leaves in *H. hainanensis*, we compared the metabolites between groups (GL vs. GU, RU vs. GU, RL vs. GL, and RL vs. RU), selected different metabolites in each group based on the screening condition that the variable importance in the projection (VIP) ≥ 1 with | Log2FC | ≥ 1, and successfully obtained 424 differentially accumulated metabolites (DAMs) in RL vs. RU and 236 DAMs in RU vs. GU, among which 189 metabolites were shared between these two comparisons (Figure 3A). We found that RU had 119 metabolites with decreased accumulation and 117 with increased accumulation compared with GU. The RL samples had 224 metabolites with decreased accumulation and 200 metabolites with increased accumulation compared with RU. The RL samples had 49 metabolites with decreased accumulation and 46 metabolites with increased accumulation compared with GL. The GL samples had 183 metabolites with decreased accumulation and 151 metabolites with increased accumulation compared with GU (Figure 3A). We further performed k-means clustering on the DAMs in the comparison group of all the samples and divided these differentially accumulated metabolites into 9 subclasses. Among the 9 subclasses 3, 8, and 9 had unique patterns of metabolites in the RU samples. The DAMs in subclasses 3 and 9 were mainly phenolic and organic acids, while subclass 8 was mainly made up of flavonoids, indicating a high accumulation of these compounds in RU relative to GU, GL, and RL (Figures 3B,C). This strongly indicates that flavonoid metabolites are the primary compounds responsible for young leaves in *H. hainanensis*.

### Transcriptome Analyses

**FIGURE 4:**
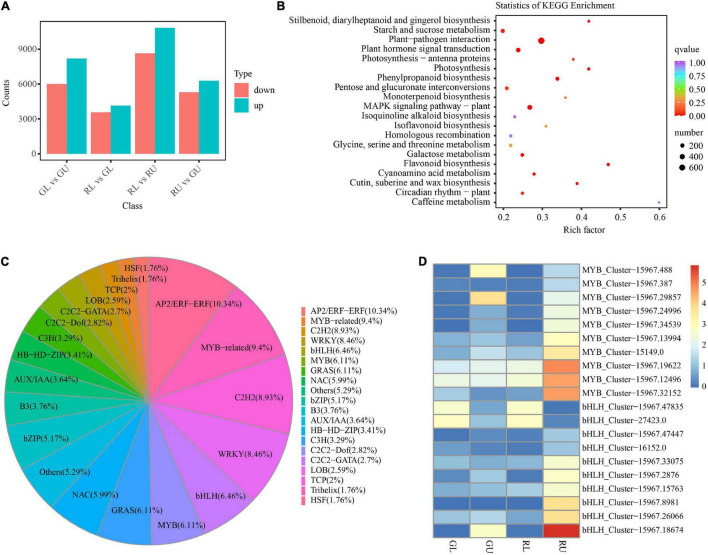
DEGs in *H. hainanensis*. **(A)** DEGs from different comparisons. **(B)** The KEGG enrichment of DEGs in RU vs. GU (*Q* values are corrected *p* values scaled by the number of comparisons, with 0 being highly significant and 1 being not significant). **(C)** Distribution of all differentially expressed transcription factors. **(D)** heatmap of the MYB and bHLH transcription factors from RU vs. GU and RL vs. RU with | log2Fold Change| ≥ 2, colors indicate log2Fpkm.

To further confirm metabolite differences between samples RU, RL, GU, and GL, RNA-seq was used to assess the differences in transcript abundance and type. A total of 86,454 transcripts were obtained by Trinity. The longest cluster sequence obtained by Corset was defined as a unigene for subsequent analysis with 83,078 such unigenes generated. Comparisons of GL vs. GU, RL vs. GL, RL vs. RU, and RU vs. GU were used to select differentially expressed genes (DEGs) with the threshold “| log2Fold Change| ≥ 1 and FDR < 0.05.” From this 14,195 DEGs (8,203 upregulated and 5,992 downregulated) from GL vs. GU; 7,718 (4,150 upregulated and 3,568 downregulated) from RL vs. GL; 19,488 (10,839 up-regulated and 8,649 downregulated) from RL vs. RU; and 11,584 (6,289 upregulated and 5,295 downregulated) from GL vs. GU were identified (Figure 4A). The RL vs. RU comparison contained the greatest number of DEGs and was further scrutinized for pigment-related pathways. The KEGG enrichment results of RU vs. GU showed that DEGs were significantly enriched in pathways related to flavonoid, stilbenoid, diarylheptanoid, and gingerol biosynthesis as well as photosynthesis (Figure 4B). Among the DEGs, the genes related to the flavonoid pathway were the most highly enriched in RU, providing additional evidence that these compounds were responsible for red leaf coloration in *H. hainanensis*.

Gene expression in some instances can be controlled by transcription factors (TFs). The TFs known as trans-acting factors, often regulate plant growth and development as well as the biosynthesis of secondary metabolites by activating or inhibiting gene expression (Latchman, 1993; Schwechheimer and Bevan, 1998). Among them, MYB and bHLH play an important role in the regulation of plant pigment (flavonoid-anthocyanin) biosynthesis (Zhuang et al., 2019). In our study, a total of 1,151 TFs were identified, with the most abundant including AP2/ERF-ERF (88), MYB-related (80), C2H2 (76), WRKY (72), bHLH (55), MYB (52), GRAS (52), NAC (51), and bZIP (44). Within the top 20 TF types, the transcription factors related to flower color in plants such as MYB accounted for 6.11% and bHLH 6.46% of all TFs (Figure 4C). Given that MYB and bHLH are known to be involved with anthocyanin accumulation, it was not surprising to find that some of these TFs were found in significantly higher abundance in RU. Specifically, three MYB TFs *HhMYB66* (Cluster-15967.19622), *HhMYB91* (Cluster-15967.12496), and *HhMYB6* (Cluster-15967.32152) and one bHLH TF *HhbHLH70* (Cluster-15967.18674) were found to be significantly upregulated in RU. The other MYB and bHLH TFs were also upregulated in RU (Figure 4D). Additionally, two bHLH TFs, Cluster-15967.47825 and Cluster-27423.0, were highly expressed in RL and GL, respectively, suggesting a putative role in leaf development (Figure 4D). Thus, increases in red leaf pigments among RU samples appears to be driven in part by the increased abundance of anthocyanin-related TFs.

### Association Analysis of Genes and Metabolites Related to Red Young Leaf Formation in *Hopea hainanensis*

**FIGURE 5:**
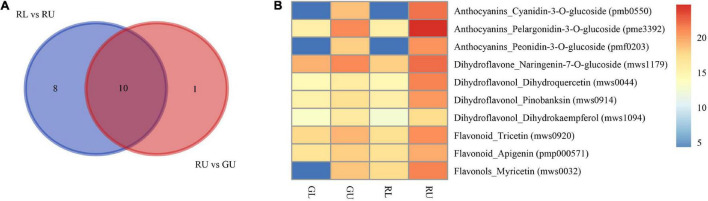
Metabolites related to red young leaves in *H. hainanensis*. **(A)** Venn diagram indicating the DAMs enriched into phenylpropanoid biosynthesis (ko00940), flavonoid biosynthesis (ko00941), and anthocyanin biosynthesis (ko00942) in the comparison of RU vs. GU and RL vs. RU. **(B)** Relative abundance of red color related metabolites in RU vs. GU and RL vs. RU indicates log2Relative abundance.

As flavonoids are the main DAMs in the red leaves of *H. hainanensis*, we selectively compared DAMs of phenylpropanoid biosynthesis (ko00940), flavonoid biosynthesis (ko00941), and anthocyanin biosynthesis (ko00942) to assess RU vs. GU and RL vs. RU. From these comparisons, 10 metabolites were found to be significantly accumulated in RU, including the anthocyanin pelargonidin-3-O-glucoside, the dihydroflavonol naringenin-7-O-glucoside, and the flavonoid myricetin as well as others in each of these three classes (Figure 5). Metabolite pme3392 was annotated as anthocyanin, pelargonidin-3-O-glucoside, which is a pigment known to produce red coloration and is found in the highest relative abundance in RUs (Figure 5B). Therefore, we suggest that this may be the most important metabolite contributing to the coloration of red young leaves.

**FIGURE 6:**
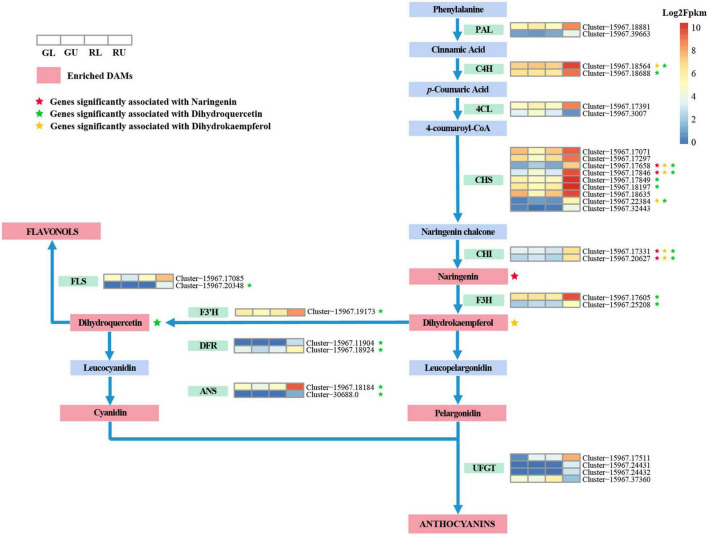
Anthocyanin biosynthetic pathway in the young red leaves of *H. hainanensis*. Metabolites, in red boxes indicate the DAMs enriched in phenylpropanoid biosynthesis (ko00940), flavonoid biosynthesis (ko00941), and anthocyanin biosynthesis (ko00942) between RU vs. GU and RL vs. RU. Heatmaps represent DEGs enriched in anthocyanin biosynthesis pathway with | log2Fold Change| ≥ 2 in the comparison of RU vs. GU and RL vs. RU. Heatmaps are ordered by GL, GU, RL, and RU from left to right, with colors indicating the log2Fpkm relative expression. Colored stars indicate a significant correlation between an increase in metabolite accumulation and an increase in the expression of an associated gene. Enzyme abbreviations: *PAL*, phenylalanine ammonium lyase; *C4H*, cinnamate-4-hydroxylase; *4CL*, 4-coumaroyl-CoA synthase; *CHS*, Chalcone synthase; *CHI*, chalcone isomerase; *F3H*, flavanone 3-hydroxylase; *F3*′*H*, flavonoid-3′-hydroxylase; *DFR*, dihydroflavonol 4-reductase; *ANS*, anthocyanidin synthase; *UFGT*, UDP-glucose flavonoid 3-O glucosyltransferase; *FLS*, flavonol synthase.

Based on the results of metabolomic analysis, anthocyanins were the main metabolites found to differ in the red young leaves of *H. hainanensis*. Thus, we focused on anthocyanin biosynthesis. DEGs associated with anthocyanin biosynthesis pathway with | log2Fold Change| ≥ 2 in comparisons of RU vs. GU and RL vs. RU were also selected for further analysis. From this, a total of 30 DEGs (Figure 6) were found, of which 2 *PAL* genes, 2 *C4H* genes, and 1 *4CL* gene, which are involved in precursor biosynthesis of flavonoids, were significantly upregulated in RU. Similarly, the anthocyanidin-associated genes, *CHS*, *CHI*, *F3H*, *F3’H*, *DFR*, and *ANS*, were found to have higher expression levels in RU. In addition, two *FLS* genes which are the key genes in the conversion of dihydroflavonols to flavonols were also significantly upregulated in RU. Interestingly, some downstream *UFGT* genes involved in the transport of anthocyanidins were downregulated in RU. When the DEGs and DAMs associated with the anthocyanin biosynthesis pathway in *H. hainanensis* were analyzed separately from the total data set, we found that among the previously screened 30 DEGs, there were 17 genes that had a significant correlation to 3 metabolites, namely dihydroquercetin (mws0044), dihydrokaempferol (mws1094), and naringenin-7-o-glucoside (mws1179), in the comparison of RU vs. GU (Pearson correlation coefficient (PCC) ≥ 0.8).

## Discussion

*Hopea hainanensis* is a valuable tree that produces high-quality wood and has numerous ecological benefits, but has become increasingly rare in recent decades, which makes the study and preservation of this species even more important. In the process of investigation and research, we found that most of the mature leaves and young leaves of *H. hainanensis* in natural forests are green, while a small minority were found to have green mature leaves and young red leaves. From breeding work, it was found that this trait was fixed and not the result of phenotypic plasticity.

From previous studies, it is well known that the red pigments in most flowers, fruits, and seeds are flavonoid compounds (Falcone Ferreyra et al., 2012). In this study, the metabolome of *H. hainanensis* leaves were comprehensively analyzed, and over 700 compounds were identified. The metabolome analyses showed that pigments, such as flavonoids were significantly accumulated in the leaf forms differently than those in young green-leaved forms. This indicated that the difference in leaf color mainly resulted from the differences in the accumulation of pigments. Leaf pigments are essential compounds in the metabolic functioning of leaves. Better understanding of the pathways by which these compounds are produced helps researchers to elucidate the evolution of plant pigments and infer their function as well as provide a blueprint for making alterations to a given pathway, resulting in selective and/or increased production of desired compounds. Chalcone synthase (*CHS*) is the first step in controlling flavonoid biosynthesis (Winkel-Shirley, 2001; Chaudhary et al., 2016), and it is the first enzyme identified in the flavonoid biosynthetic pathway. *CHS* is an important regulatory gene located upstream in the flavonoid biosynthesis pathway, and its overexpression may positively affect the expression of downstream *CHI* genes that affect the production of flavonoids (Kreuzaler and Hahlbrock, 1972; Awasthi et al., 2016). Here we saw the upregulation of *CHS*, which is accompanied by the upregulation of *CHI* and its downstream counterpart naringenin. In the anthocyanin biosynthetic pathway, dihydrokaempferol is a key intermediate product that can be converted to dihydroquercetin by the enzyme *F3’H*, and/or converted to dihydromyricetin by *F3’5’H* (Falcone Ferreyra et al., 2012). Here, we found that two *F3H* genes were upregulated, and dihydrokaempferol increased. An *F3’H* gene was found to be highly expressed along with increases in the accumulation of the direct downstream product in the red young leaves. This result is similar to a study that determines the pathway by which red longan (*Dimocarpus longan*) fruits were produced. Yi et al. (2021) revealed that genes related to enzymes leading up to dihydrokaempferol were significantly upregulated in red pericarp longan fruits. While the pathways to produce red pigments might be similar between these two distantly related species, the timing for activating them is quite different as the pathway is initiated in young leaves of *H. hainanensis* and in maturing fruits of longan. Apart from these intermediate products mentioned above, their downstream genes and products were also upregulated in *H. hainanensis*. On the whole, we found *C4H*, *CHS*, *CHI*, *F3H*, *F3’H*, *DFR*, *ANS*, and *FLS* were positively correlated with differentially accumulated metabolic intermediates in the comprehensive analysis of the metabolome and transcriptome, and these genes possessed significantly higher expression levels, which is consistent with results in the purple-leaved Jujube (Li et al., 2021). These results suggest that the high expression of *C4H*, *CHS*, *CHI*, *F3H*, and *F3’H* could promote the accumulation of dihydroquercetin (mws0044) and dihydrokaempferol (mws1094). The accumulation of these two metabolites will recruit and promote the expression of downstream genes, such as *DFR*, *ANS*, and *FLS*, to further generate downstream products. In addition, we found that in the anthocyanin biosynthesis of RU vs. RL, some *UFGT* genes (cluster-15967.49721) were negatively correlated with pelargonidin-3-o-glucoside (−0.831) as the log2Fold Change of this gene in RU vs. GU was −1.83. Downregulation of some *UFGT* genes was also found in red pericarp longan fruits (Yi et al., 2021) and thought to be involved with increased anthocyanin accumulation. Furthermore, the high expression of *FLS* and the accumulation of the flavonoid apigenin (pmp000571) also revealed that flavonoids, in addition to anthocyanins, pelargonidin, and cyanidin, are also responsible for the red color of young red-leaved lines.

Flavonoid biosynthesis genes are often regulated by the interaction of transcription factors in different gene families. In monocotyledons and dicotyledons, genes involved in the anthocyanin biosynthesis pathway are differentially regulated by R2R3-MYB transcription factors, bHLH, and WD40 proteins (Grotewold, 2005; Petroni and Tonelli, 2011; Xu et al., 2015; Zhuang et al., 2019). Therefore, the combination of R2R3-MYB, bHLH, and WD40 transcription factors and their interactions (MYB-bHLH-WD40 complex) determine the activation and spatio-temporal expression of anthocyanin synthesis structural genes (Petroni and Tonelli, 2011). Unsurprisingly, our results also showed similarities to other studies in apple and grape (Walker et al., 2007; An et al., 2020), wherein the TFs, MYB and bHLH, are the key regulators of anthocyanin biosynthesis and accumulation. The MYB and bHLH transcription factors are present in all eukaryotes and are the two largest families of plant transcription factors (Feller et al., 2011). More than 20 years ago, the first transcription factor encoding proteins in the MYB domain required for anthocyanin synthesis was discovered in plants (Paz-Ares et al., 1987). Since then more studies have found that MYB transcription factors, such as some R2R3-MYBs, including AtMYB75/PAP1, AtMYB90/PAP2, AtMYB113, and AtMYB114 that control anthocyanin biosynthesis in vegetative tissues play an important role in flavonoid biosynthesis (Gonzalez et al., 2008). As for the bHLH transcription factors, the abnormal expression of *bHLH3* in mulberry fruits was shown to disrupt the balance of the flavonoid metabolic network, leading to changes in the content and proportion of anthocyanins, flavonoids, and flavonols in the different colored mulberry fruits (Li et al., 2020). In this process, MYB transcription factors that are mainly involved in the regulation of flavonoid biosynthetic genes, for example, MYB transcription factors SbY1 in *Sorghum bicolor*, may have an impact on the expression of *CHS*, *CHI*, and *DFR*, resulting in regulation of 3-deoxyflavonoid biosynthesis (Du et al., 2009). In *Solanum tuberosum*, StD is thought to regulate the *F3H*, *DFR*, and *F3’5’H* genes, and thus play a role in the regulation of anthocyanin biosynthesis (Jung et al., 2009). In our study, we also found that three MYB TFs *HhMYB66* (Cluster-15967.19622), *HhMYB91* (Cluster-15967.12496), and *HhMYB6* (Cluster-15967.32152), and one bHLH TF *HhbHLH70* (Cluster-15967.18674) were significantly in higher abundance in RU. Moreover, *CHS*, *CHI*, *F3H*, *F3’H*, and *DFR* were also upregulated, implying that these genes may be the key genes regulating flavonoid/anthocyanin synthesis in *H. hainanensis* red leaves.

By combining the results of metabolome and transcriptome data we suggest that there are two main anthocyanin biosynthesis pathways after branching from dihydrokaempferol, and the significant upregulation of genes in these pathways leads to an increased accumulation of anthocyanin and flavonoid derivatives, which together are responsible for the young red leaves of *H. hainanensis*. Now, the red pigments responsible for young red leaves have been identified, while the follow-up work examining the simultaneously upregulated genes related to stilbenoid production and photosynthesis (found in this study) needs to be conducted to uncover potentially synergistic roles of these phytochemicals in leaf metabolism.

## Materials and Methods

### Plant Materials

During the investigation and cultivation of *H. hainanensis*, we found that most *H. hainanensis* have green mature and young leaves, whereas a small number of individuals possess mature green leaves and young red leaves. After we collected seeds and raised seedlings individually (the seedlings from one tree’s seeds are regarded as a line), it was found that their offspring had the same leaf-color, and this pattern was maintained after reproduction, which indicated that genetic differences controlled the leaf color trait. To investigate the metabolomic and transcriptomic differences between the red and green leaf lines, we chose one red leaf individual (referred to as Line 5 in our lab breeding nomenclature) and one green leaf pedigree (referred to as Line 10) for further analyses. The red-leafed individuals were found during the course of forest surveys. Both red and green individuals have been grown from seeds for 2 years in the outdoor common garden at the Research Institute of Tropical Forestry (Chinese Academy of Forestry).

Next, we selected three independent samples from each pedigree to represent the biological duplications (referred to as Red1, Red2, and Red3; Green1, Green2, and Green3). To optimize variation among red and green leaves, we sampled the leaves along the axis of the stem from the 2nd expanded leaf (young leaves) to the 6th unfolded leaf (mature leaves) with a total of 12 samples. Duplicated samples from young red-leaved lines (RU), green mature leaves in red lines (RL), young green leaves in green lines (GU), and green mature leaves in green lines (GL) were harvested and stored at −80°C after snap-freezing with liquid nitrogen.

### UPLC-MS/MS Instrumentation and Analyses

The freeze-dried leaves were crushed using a mixer mill (MM 400, Retsch, Haan, German) with a zirconia bead for 1.5 min at 30 Hz. From this 100 mg powder was weighed and extracted overnight at 4°C with 1.2 mL 70% aqueous methanol. Following centrifugation at 12,000 rpm for 10 min, the supernatant was removed and syringe-filtered (SCAA-104, 0.22μm pore size; ANPEL, Shanghai, China)^1^ before UPLC-MS/MS analysis.

The sample extracts were analyzed using a UPLC-ESI-MS/MS system (UPLC, SHIMADZU Nexera X2^2^; MS, Applied Biosystems 4500 Q TRAP^3^). The analytical conditions were as follows for UPLC: column, Agilent SB-C18 (1.8 μm, 2.1 mm*100 mm); the mobile phase consisted of solvent A, pure water with 0.1% formic acid, and solvent B, acetonitrile with 0.1% formic acid. Sample measurements were performed with a gradient program that employed the starting conditions of 95% A, 5% B. Within 9 min, a linear gradient to 5% A, 95% B was programmed, and a composition of 5% A, 95% B was kept for 1 min. Subsequently, a composition of 95% A, 5% B was adjusted within 1.10 min and kept for 2.9 min. The column oven was set to 40°C and the injection volume was 4 μL. The effluent was injected into an ESI-triple quadrupole-linear ion trap (QTRAP)-MS.

The mass spectrometry analysis followed the method of Chen et al. (2013). Linear ion trap (LIT) and triple quadrupole (QQQ) scans were acquired on a triple quadrupole-linear ion trap mass spectrometer (Q TRAP), AB4500 Q TRAP UPLC/MS/MS system, equipped with an ESI Turbo Ion-Spray interface and operating in positive and negative ion modes and controlled by Analyst 1.6.3 software (AB Sciex). The ESI source operation parameters were as follows: ion source turbo spray; source temperature 550°C; ion spray voltage (IS) 5,500 V (positive ion mode)/-4,500 V (negative ion mode); ion source gas I (GSI), gas II (GSII), and curtain gas (CUR) were set at 50, 60, and 25.0 psi, respectively; the collision gas (CAD) was set to high. Instrument tuning and mass calibration were performed with 10 and 100 μmol/L polypropylene glycol solutions in QQQ and LIT modes, respectively. The QQQ scans were acquired as MRM experiments with collision gas (nitrogen) set to medium. DP and CE for individual MRM transitions were done further with DP and CE optimization. A specific set of MRM transitions were monitored for each period according to the metabolites eluted within this period (Falcone Ferreyra et al., 2012).

Unsupervised PCA (principal component analysis) (Chen et al., 2009) was performed by statistics function prcomp within R^4^. The data was normalized before unsupervised PCA. The hierarchical cluster analysis (HCA) results of samples and metabolites were presented as heatmaps with dendrograms, while the Pearson correlation coefficients (PCC) between samples were calculated by the cor function in R and presented as heatmaps. Both HCA and PCC were carried out by R package pheatmap^5^. For HCA, normalized signal intensities of metabolites (unit variance scaling) are visualized as a color spectrum. Significantly accumulated metabolites between groups were determined by VIP ≥ 1 and absolute Log2FC (fold change) ≥ 1. VIP values were extracted from orthogonal partial least squares discriminant analysis (OPLS-DA) results, which also contain score plots and permutation plots, and was generated using R package MetaboAnalystR (Chong and Xia, 2018). Data were log-transformed (log2) and mean centered before OPLS-DA. To avoid overfitting, a permutation test (200 permutations) was performed. Identified metabolites were annotated using the KEGG compound database^6^ (Kanehisa and Goto, 2000) and the annotated metabolites were then mapped to the KEGG pathway database^7^. Pathways with significantly accumulated metabolites were then fed into metabolite sets enrichment analysis (MSEA), with significance determined by *p*-values from hypergeometric tests.

### RNA Sequencing and Differentially Expressed Genes Analysis

Total RNA was extracted from the young and mature leaves of *H. hainanensis* red and green lines. RNA-Seq was performed by Biomarker Technologies Co., Ltd. (Beijing, China). Sequencing libraries were generated using the NEBNext^®^UltraTM RNA Library Prep Kit for Illumina^®^ (New England Biolabs, Ipswich, MA, United States) following the manufacturer’s recommendations. Sample-specific indices were added during library preparation such that sequencing could be multiplexed. The library preparations were sequenced on an Illumina HiSeq X platform (Illumina, Inc., San Diego, CA, United States).

After filtering and quality control of the raw sequence data, clean reads were *de novo* assembled by Trinity v2.6.6 (Grabherr et al., 2011; Haas et al., 2013), and Corset v 1.07 (Davidson and Oshlack, 2014) was used to perform hierarchical clustering of the transcripts by comparing the number of reads and expression patterns of the transcripts. The transcript sequences obtained by Trinity were used as the reference sequence, and the longest Cluster sequence obtained by Corset hierarchical clustering was used as a unigene for subsequent analysis. To conduct the gene function annotation, the unigene sequences were compared with KEGG, NR, Swiss-Prot, GO, COG/KOG, and Trembl databases using the BLAST software (Altschul et al., 1990). Then the amino acid sequences predicted from unigenes were compared with Pfam database by HMMER software (Mistry et al., 2013) to obtain unigene annotation information. Plant-transcription factor prediction was performed using iTAK software (Zheng et al., 2016), which integrates PLNTFDB and PLANTTFDB with TFs identified by hmmscan.

The transcriptome assembled by Trinity was used as a reference sequence, and the clean reads of each sample were mapped on the reference to calculate the mapping rate of each sample with bowtie2 in RSEM. DESeq2 v1.22.2 (Varet et al., 2016) was used to obtain the DEGs between any two biological conditions. After that the Benjamini-Hochberg method was used to conduct multiple hypothesis testing correction on the hypothesis test probability (*p*-value) to obtain the false discovery rate (FDR). DEGs were selected under the conditions of | log2Fold Change| ≥ 1 and FDR < 0.05. Finally, we analyzed the KEGG pathway enrichment in all DEGs and drew the corresponding network regulation pathway map.

## Conclusion

Integrated metabolome and transcriptome analyses of young and mature leaves of red and green lines of *H. hainanensis* revealed that the accumulation of anthocyanins and flavonoids in RU was significantly higher in red samples than in the three green samples. The main color-related products in red leaves were anthocyanins and flavonoids, among which pelargonidin and cyanidin were the most important metabolites. Transcriptome results showed that the key genes in the anthocyanin pathway in RU were expressed at significantly higher levels compared to green-leaved samples, leading to the greater accumulation of red-colored metabolites. In addition to the structural genes in the anthocyanin/flavonoid pathway, the TFs MYB and bHLH were highly expressed in RU over the three green-leaved samples. Three MYBs *HhMYB66* (Cluster-15967.19622), *HhMYB91* (Cluster-15967.12496), and *HhMYB6* (Cluster-15967.32152), and one bHLH *HhbHLH70* (Cluster-15967.18674) were expressed at significantly higher levels in RU. As such these four TFs are likely essential to initiate the pathway associated with increased anthocyanin and flavonoids found in RU. While the anthocyanin pathway in *H. hainanensis* is similar to others described from distantly related plant lineages, it is also different in several aspects, such as the absence of delphinidin derivatives. The anthocyanin/flavonoid pathway described here provides a valuable resource for the study of leaf pigment evolution as well as in the development of red-leaved cultivars for use in horticultural and forestry applications.

## Data Availability Statement

The datasets presented in this study can be found in online repositories. The names of the repository/repositories and accession number(s) can be found below: NCBI; PRJNA795198.

## Author Contributions

GH designed and supervised implementation of the studies. GL and GY were responsible for sampling. XL and QH supervised the statistical analyses, constructed the tables, and wrote the manuscript. ZZ and KL cultivated the seedlings of *H. hainanensis*. XW and ZW carried out all technical aspects and crafted the final version. LT provided suggestions on various aspects of the study and helped edit the manuscript. All authors contributed to the article and approved the submitted version.

## Conflict of Interest

The authors declare that the research was conducted in the absence of any commercial or financial relationships that could be construed as a potential conflict of interest.

## Publisher’s Note

All claims expressed in this article are solely those of the authors and do not necessarily represent those of their affiliated organizations, or those of the publisher, the editors and the reviewers. Any product that may be evaluated in this article, or claim that may be made by its manufacturer, is not guaranteed or endorsed by the publisher.

## Footnotes

1
http://www.anpel.com.cn

2
www.shimadzu.com.cn

3
www.appliedbiosystems.com.cn

4
www.r-project.org

5
https://cran.r-project.org/web/packages/pheatmap/index.html

6
http://www.kegg.jp/kegg/compound

7
http://www.kegg.jp/kegg/pathway.html
